# Asymptomatic Atrial Fibrillation in Contemporary Clinical Practice: Insights from the REGUEIFA Registry

**DOI:** 10.3390/medsci14030401

**Published:** 2026-07-18

**Authors:** Carlos Minguito-Carazo, Laila González Melchor, María Vázquez Caamaño, Emilio Fernández-Obanza Windcheid, Raquel Marzoa, Miriam Piñeiro Portela, Eva González Babarro, Pilar Cabanas Grandío, Olga Durán Bobín, Óscar Prada Delgado, Juliana Elices Tejada, Evaristo Freire, Mario Gutiérrez Feijoo, Javier Muñiz, Francisco Gude, Eduardo Barge Caballero, Carlos González-Juanatey, Javier García Seara

**Affiliations:** 1 Department of Cardiology, Hospital Universitario de Santiago de Compostela, 15706 Santiago de Compostela, Spain; 2 Cellular and Molecular Cardiology Unit, Health Research Institute of Santiago de Compostela (IDIS-SERGAS), 15706 Santiago de Compostela, Spain; 3 Centro de Investigación Biomédica en Red de Enfermedades Cardiovasculares (CIBERCV), Instituto de Salud Carlos III, 28029 Madrid, Spain; 4 Department of Cardiology, Hospital Universitario Lucus Augusti, Health Research Institute of Santiago (IDIS), 27001 Lugo, Spain; 5 Department of Cardiology, Hospital San Rafael, 15006 A Coruña, Spain; 6 Department of Cardiology, Hospital Álvaro Cunqueiro and Health Research Institute of Southern Galicia (IISGS), 36312 Vigo, Spain; 7 Department of Cardiology, Hospital Arquitecto Marcide, 15405 Ferrol, Spain; 8 Department of Cardiology, Biomedical Research Institute of A Coruña (INIBIC), Hospital Universitario de A Coruña, 15006 A Coruña, Spain; 9 Department of Cardiology, Hospital Universitario Montecelo, 36164 Pontevedra, Spain; 10 Department of Cardiology, Hospital Universitario de Ourense, 32003 Ourense, Spain; 11 Cardiovascular Research Group, Department of Health Sciences and Biomedical Research Institute of a Coruña (INIBIC), University of A Coruña, CIBERCV, 15006 A Coruña, Spain; 12 Research Methodology Group, Health Research Institute of Santiago (IDIS), University of Santiago de Compostela (USC), 15706 Santiago de Compostela, Spain

**Keywords:** atrial fibrillation, EHRA classification, antiarrhythmic therapy

## Abstract

**Background**: Asymptomatic atrial fibrillation (AF) represents a substantial proportion of diagnosed AF cases; however, its prognostic implications remain controversial. This study aimed to compare clinical characteristics, treatment strategies, and outcomes between asymptomatic and symptomatic AF patients in a contemporary real-world registry. **Methods**: This observational subanalysis included 997 patients from the prospective multicenter REGUEIFA registry with 2-year follow-up. Patients were classified as asymptomatic if they had a baseline European Heart Rhythm Association (EHRA) score of I. Clinical characteristics, therapeutic strategies, and the incidence of a composite endpoint including cardiovascular death, stroke, major bleeding, or worsening heart failure were analyzed. **Results:** Overall, 33.7% of patients were asymptomatic. Compared with symptomatic patients, asymptomatic patients had a lower prevalence of heart failure, hypertension, and cardiomyopathy, lower CHA_2_DS_2_-VASc scores, and a higher prevalence of preserved left ventricular ejection fraction. Rhythm control strategies were less frequently used in asymptomatic patients (58.33% vs. 65.96%; *p* = 0.018), despite similar arrhythmia recurrence rates after treatment (*p* = 0.490). The incidence of the composite endpoint was lower in asymptomatic patients (8.39 [6.53–10.78] vs. 12.12 [10.43–14.09] per 100 person-years; *p* = 0.013), mainly driven by lower cardiovascular mortality. No significant differences were observed in stroke, major bleeding, worsening heart failure, or all-cause mortality. In multivariable analysis adjusted for clinically relevant covariates, symptomatic patients exhibited a higher incidence of the composite endpoint. **Conclusions:** In this contemporary real-world registry, asymptomatic AF patients exhibited a lower burden of cardiovascular comorbidities and lower cardiovascular mortality than symptomatic patients, while rates of stroke, heart failure worsening, major bleeding, and arrhythmia recurrence were similar. These findings suggest that prognosis is influenced predominantly by baseline comorbidity burden, and the independent contribution of symptom status itself, although statistically significant, appears modest and should be interpreted with caution given the observational design.

## 1. Introduction

Atrial fibrillation (AF) is the most common sustained arrhythmia in clinical practice, with a steadily increasing prevalence worldwide, largely driven by population aging and the growing burden of cardiovascular comorbidities. In Spain, AF is estimated to affect 4.4% of the adult population, exceeding 10% among individuals older than 75 years [1,2]. Although AF may present with a wide range of symptoms—from palpitations and dyspnea to fatigue or syncope—several studies have shown that between 27% and 40% of patients are asymptomatic at the time of diagnosis. This proportion may be even higher when silent episodes detected through prolonged monitoring, implantable devices, or systematic screening strategies are taken into account [3,4]. This clinical heterogeneity poses important challenges for individualized patient management.

Since the publication of the EAST-AFNET 4 trial, current clinical practice guidelines support rhythm control strategies particularly in symptomatic patients, aiming not only to improve symptoms but also to reduce cardiovascular mortality and hospitalizations [5,6,7]. However, most randomized clinical trials supporting these recommendations have predominantly included patients with symptomatic AF, leading to a less structured approach for asymptomatic cases. This has resulted in considerable variability in clinical practice and limited guidance, particularly regarding the role of rhythm control strategies and the optimal intensity of follow-up. Moreover, uncertainty persists as to whether the absence of symptoms truly reflects a more benign prognosis or may instead mask more advanced or persistent forms of the disease, especially when diagnosis is delayed.

In this context, the REGUEIFA registry (Galician Registry of Atrial Fibrillation) was established as a national multicenter initiative aimed at providing a comprehensive characterization of patients with AF managed in cardiology outpatient clinics in Spain [8]. The present subanalysis compares clinical characteristics, treatment strategies, and the incidence of clinical events during follow-up between patients with asymptomatic AF (EHRA I) and those with any degree of symptoms (EHRA II–IV), with the objective of clarifying the prognostic profile of asymptomatic AF and contributing to the optimization of its clinical management.

## 2. Materials and Methods

### 2.1. Study Design and Population

A prospective, multicenter observational study was conducted using data from the REGUEIFA registry, developed within the setting of routine clinical practice in hospital-based and outpatient cardiology clinics. Patients with a diagnosis of atrial fibrillation (AF), either newly diagnosed or previously known, who were evaluated by cardiology specialists were included. Clinical events were collected prospectively and adjudicated by the treating physicians at each participating center according to predefined registry definitions.

Patients were classified into two groups according to their symptomatic profile at the time of inclusion using the European Heart Rhythm Association (EHRA) classification: an asymptomatic group (EHRA I) and a symptomatic group (EHRA IIa, IIb, III, or IV). In addition to the EHRA classification, the EQ-5D quality-of-life questionnaire was used to provide a more comprehensive assessment of functional status. Clinical follow-up was performed prospectively at three time points: baseline (inclusion), 1 year, and 2 years. The study was conducted in accordance with the Declaration of Helsinki and approved by the Ethics Committee of Santiago de Compostela/Lugo.

### 2.2. Study Objectives

The primary objective was to compare the overall incidence and event-free survival of the composite endpoint of cardiovascular death, ischemic stroke, major bleeding, or worsening heart failure (HF) between symptomatic and asymptomatic patients with AF over a 2-year follow-up period. Worsening HF was defined as progression to more advanced New York Heart Association (NYHA) functional classes compared with baseline or the need for hospitalization due to decompensated HF.

Secondary objectives included the evaluation of differences in incidence rates and event-free survival for all-cause mortality, cardiovascular mortality, stroke, major bleeding, and worsening HF. Additional secondary objectives were to assess differences in therapeutic strategies regarding rhythm control between both groups and to compare AF recurrence rates among patients who were restored to sinus rhythm and managed with a rhythm control strategy.

### 2.3. Statistical Analysis

Continuous variables are presented as mean ± standard deviation (SD) and were compared using Student’s t test or the Mann–Whitney U test, as appropriate based on data distribution. Categorical variables are expressed as percentages and were compared using the chi-square test or Fisher’s exact test, as appropriate. Incidence rates per 100 person-years and their 95% confidence intervals at 2 years were calculated using the quadratic approximation to the log-likelihood of the Poisson distribution. Survival curves from the initial diagnosis of AF were constructed using the Kaplan–Meier method and compared using the log-rank test. Multivariable Cox proportional hazards regression analysis was performed to evaluate the association between symptomatic status and the composite endpoint, adjusting for sex, hypertension, cardiomyopathy, CHA_2_DS_2_-VASc score, and LVEF ≤ 40%, selected based on their association with symptomatic status and with the study outcomes. Baseline HF was not included as a covariate in this model, since “worsening HF” is itself a component of the composite endpoint and is derived from baseline HF status; adjusting for baseline heart failure would therefore introduce conceptual circularity into the model. Statistical significance was defined as a two-sided *p* value < 0.05. All analyses were performed using STATA version 15.1.

## 3. Results

### 3.1. Study Population

A total of 997 patients were included in the study (mean age 67.61 ± 11.98 years; 32.20% women). Of these, 336 patients (33.70%) were classified as EHRA I (asymptomatic), while 661 patients (66.30%) presented with symptoms at the time of registry inclusion (389 [39.02%] EHRA IIa, 170 [17.05%] EHRA IIb, 95 [9.53%] EHRA III, and 7 [0.70%] EHRA IV). The proportion of women was lower in the asymptomatic group compared with the symptomatic group (26.79% vs. 34.95%; *p* = 0.009).

Symptomatic patients more frequently had a history of heart failure (10.12% vs. 17.25%; *p* = 0.003), cardiomyopathy (4.46% vs. 9.53%; *p* = 0.005), and hypertension (54.46% vs. 66.41%; *p* < 0.001). In addition, symptomatic patients had higher CHA_2_DS_2_-VASc scores (2.17 ± 1.57 vs. 2.46 ± 1.51; *p* = 0.003). A higher proportion of patients with preserved left ventricular ejection fraction (>50%) was observed in the asymptomatic group (85.58% vs. 75.32%; *p* = 0.003), with no significant differences in the prevalence of major valvular heart disease.

At study inclusion, the main reason for consultation in the overall cohort was AF (81.1%), with 377 patients (37.8%) representing a first diagnosis. Among patients with previously diagnosed AF, 64.7% were managed with a rhythm control strategy, which was more frequently adopted in symptomatic patients (58.9% vs. 67.4%; *p* = 0.029). Regarding AF subtype, permanent AF was more prevalent among asymptomatic patients than symptomatic patients (31.55% vs. 26.93%; *p* < 0.001). A higher proportion of first AF diagnoses was also observed in the asymptomatic group (44.35% vs. 34.49%; *p* = 0.002). Baseline characteristics of the cohort are summarized in Table 1.

### 3.2. Evolution of AF Subtype and Symptoms

During follow-up, a progressive increase in the proportion of asymptomatic patients (EHRA I) was observed, rising from 33.70% at baseline to 61.46% at 1 year and 66.97% at 2 years. With respect to AF subtype, no significant differences were found between groups in the proportion of patients who experienced changes in AF subtype during follow-up (30.80% vs. 37.52%; *p* = 0.072).

### 3.3. Quality of Life

Quality of life was assessed at baseline using the EQ-5D questionnaire. Compared with symptomatic patients, asymptomatic patients reported a lower prevalence of mobility problems (22.08% vs. 35.31%; *p* < 0.001), difficulties with usual activities (14.94% vs. 26.97%; *p* < 0.001), pain or discomfort (25.65% vs. 33.23%; *p* = 0.018), and anxiety or depression (23.70% vs. 33.39%; *p* < 0.001) (Table 2).

### 3.4. Primary Endpoint

Over a median follow-up of 2.08 years (interquartile range 1.96–2.43), 76 patients (7.62%) died, including 35 deaths (3.51%) of cardiovascular cause. Thirteen patients (1.30%) experienced an ischemic stroke, 159 (15.95%) developed worsening heart failure, and 71 (7.10%) suffered major bleeding events. Overall, 231 patients (23.17%) experienced the composite endpoint.

**Figure 1:**
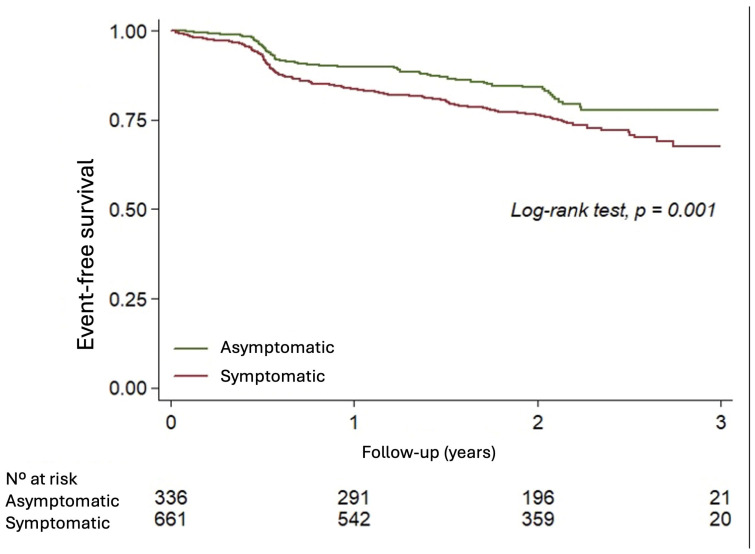
Event-free survival for the composite endpoint of cardiovascular death, worsening heart failure, stroke, and major bleeding.

The incidence rate of the primary composite endpoint of cardiovascular death, stroke, worsening heart failure, or major bleeding was significantly lower in asymptomatic than in symptomatic patients (8.39 [6.53–10.78] vs. 12.12 [10.43–14.09] per 100 person-years; *p* = 0.013) (Table 3). Event-free survival was also higher among asymptomatic patients (log-rank *p* = 0.001) (Figure 1). In multivariable analysis, symptomatic status remained associated with a higher incidence of the composite endpoint after multivariable adjustment (HR 1.40, 95% confidence interval 1.02–1.91; *p* = 0.036) (Table 4).

### 3.5. Secondary Endpoints

**Figure 2:**
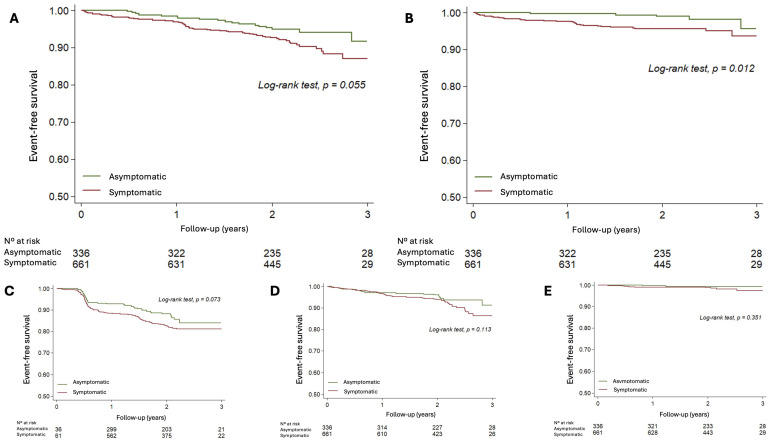
Event-free survival for all-cause mortality (**A**), cardiovascular mortality (**B**), worsening heart failure (**C**), stroke (**D**), and major bleeding (**E**).

No significant differences were observed in the incidence rate of all-cause mortality between groups (2.50 [1.60–3.90] vs. 4.14 [3.20–5.40] per 100 person-years; *p* = 0.055), nor in overall survival (log-rank *p* = 0.055), although a trend toward a higher number of events was noted in symptomatic patients. In contrast, symptomatic patients showed a significantly higher incidence rate of cardiovascular mortality (0.69 [0.29–1.65] vs. 2.14 [1.50–3.06] per 100 person-years; *p* = 0.013) and lower cardiovascular survival (log-rank *p* = 0.012). No significant differences were found between groups in the incidence rates or event-free survival for stroke, major bleeding, or worsening heart failure (Table 4, Figure 2).

### 3.6. Therapeutic Management of AF

After registry inclusion, symptomatic patients were more frequently treated with a rhythm control strategy compared with asymptomatic patients (65.96% vs. 58.33%; *p* = 0.018). A higher rate of sinus rhythm restoration after the first intervention was also observed in symptomatic patients (54.64% vs. 47.32%; *p* = 0.044). Electrical cardioversion was the most commonly used strategy to restore and maintain sinus rhythm (24.17% of the cohort), followed by catheter ablation (16.75%) and pharmacological cardioversion (2.61%).

Compared with symptomatic patients, asymptomatic patients underwent electrical cardioversion (19.35% vs. 26.63%; *p* = 0.011) and catheter ablation (5.95% vs. 22.24%; *p* < 0.001) less frequently. In parallel, symptomatic patients were more often treated with antiarrhythmic drugs (45.08% vs. 26.49%; *p* < 0.001), beta-blockers (71.10% vs. 62.50%; *p* = 0.006), and diuretics (38.43% vs. 26.49%; *p* < 0.001), reflecting a more intensive management approach in this group.

### 3.7. AF Recurrence

**Figure 3:**
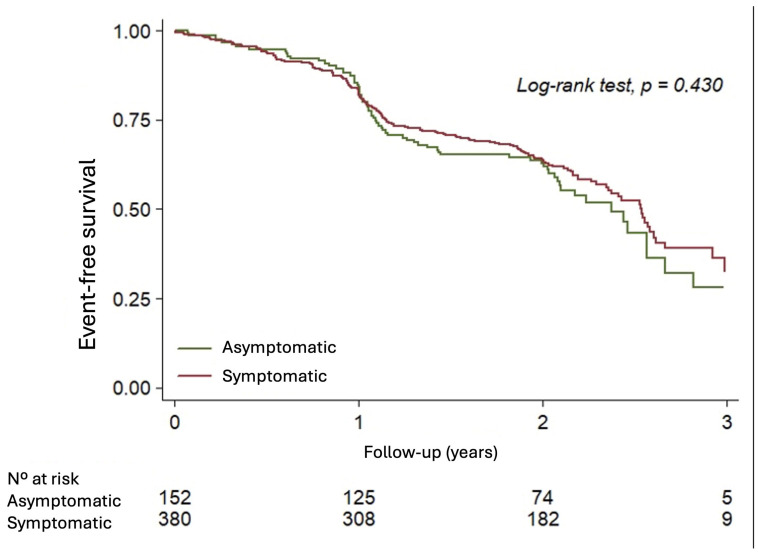
Event-free survival for atrial fibrillation recurrence.

Among the 532 patients who were in sinus rhythm after the first visit or in whom an active rhythm control strategy was implemented, AF recurrence rates during follow-up were similar between asymptomatic and symptomatic patients (46.71% vs. 43.42%; *p* = 0.490). No significant differences were observed in flutter- or AF-free survival over time (*p* = 0.430) (Figure 3).

## 4. Discussion

This prospective multicenter study evaluated the association between AF-related symptoms and the occurrence of clinical events in a multicenter real-world registry. The main findings can be summarized as follows: (1) symptomatic patients exhibited a higher incidence of the composite endpoint of cardiovascular death, stroke, major bleeding, or worsening heart failure (HF); (2) this difference was mainly driven by a higher rate of cardiovascular mortality among symptomatic patients; (3) however, no differences were observed in the incidence or event-free survival for all-cause mortality, stroke, major bleeding, or worsening HF according to symptom status; (4) asymptomatic patients were less likely to receive a rhythm control strategy; and (5) among patients treated with rhythm control, AF recurrence rates did not differ according to the presence or absence of symptoms.

Traditionally, asymptomatic AF has been considered a condition with a more favorable prognosis, partly because of its association with more paroxysmal forms and the absence of immediate clinical impact [9].

However, this concept has been challenged by several contemporary studies. A recent meta-analysis including 36 observational studies and 217,850 patients found no differences between symptomatic and asymptomatic patients in the risk of all-cause mortality, cardiovascular mortality, thromboembolism, stroke, hospitalization, or myocardial infarction [10]. Symptomatic patients did, however, show a higher incidence of new-onset HF (HR 1.33) and a lower progression to permanent AF (HR 0.70), findings that may reflect more aggressive therapeutic strategies and closer follow-up in this subgroup.

Similarly, Boriani et al. reported no differences at 2 years in the composite endpoint of all-cause mortality and major adverse cardiovascular events (cardiovascular death, thromboembolism, or acute coronary syndrome) between asymptomatic (EHRA I) and symptomatic (EHRA ≥ II) patients [11]. Nevertheless, a particularly high-risk subgroup was identified: asymptomatic patients with left ventricular ejection fraction ≤ 40%, who experienced the highest event rates, even exceeding those of symptomatic patients with ventricular dysfunction. These observations are consistent with prior registries such as EORP-AF, in which asymptomatic patients more frequently had permanent AF, a higher burden of comorbidities, and consequently higher 1-year mortality than symptomatic patients (9.4% vs. 4.2%) [4].

Although some of these findings may be influenced by selection bias or differences in patient capture, they underscore the marked heterogeneity of asymptomatic AF. While some studies suggest similar risks regardless of symptoms, others associate asymptomatic AF with higher mortality, supporting the notion that asymptomatic AF warrants careful clinical evaluation [12]. In our study, asymptomatic patients exhibited a lower incidence of the composite endpoint of cardiovascular death, stroke, major bleeding, or worsening HF, mainly driven by lower cardiovascular mortality. Although this finding may appear discordant with some previous reports, it is likely explained by a more favorable baseline profile in this subgroup, including a lower prevalence of hypertension, HF, and cardiomyopathy, higher left ventricular ejection fraction, and lower CHA_2_DS_2_-VASc scores. These factors are well-established predictors of cardiovascular mortality and may have substantially influenced the differences observed between groups. In this regard, AF-related symptoms may not exclusively reflect arrhythmia burden itself, but also the presence of concomitant structural heart disease, HF, or other cardiovascular comorbidities contributing to worse outcomes. Accordingly, the lower cardiovascular mortality observed in asymptomatic patients should not be interpreted as a protective effect of asymptomatic status itself, but rather as a reflection of the lower baseline comorbidity burden in this subgroup. Although symptomatic status remained associated with the composite endpoint after multivariable adjustment, residual confounding cannot be excluded given the observational nature of the study and the absence of propensity score matching or sensitivity analyses excluding patients with heart failure or reduced left ventricular ejection fraction.

It should also be noted that symptom status was classified according to the baseline EHRA score, whereas this classification proved to be highly dynamic during follow-up: the proportion of asymptomatic patients increased from 33.70% at baseline to 61.46% at 1 year and 66.97% at 2 years. This pattern suggests that a substantial number of patients initially classified as symptomatic became asymptomatic over time, likely reflecting symptomatic improvement after treatment, changes in rhythm status, or adaptation to the arrhythmia. Because our analysis relied on a fixed baseline classification, it primarily captures the prognostic impact of the initial symptom status and does not account for this evolving trajectory. Future studies incorporating time-updated symptom assessment would help clarify whether the observed associations are driven by baseline status, its evolution over time, or both.

Importantly, the incidence of the composite endpoint among asymptomatic patients was not negligible (8.39 per 100 person-years), and no differences were observed when individual outcomes—such as all-cause mortality, stroke, major bleeding, or worsening HF—were analyzed separately. These results are consistent with findings from a subanalysis of the GARFIELD-AF registry including more than 52,000 patients (25% asymptomatic), which showed no significant differences in all-cause mortality, stroke, or bleeding [13]. Similarly, in the study by Lee et al., including over 10,000 patients of whom 42% had asymptomatic AF, EHRA class I was not associated with lower all-cause mortality or a reduced incidence of a composite endpoint including death, stroke, transient ischemic attack, systemic embolism, myocardial infarction, or HF [14]. In line with these data, the ORBIT-AF registry also found no differences in all-cause mortality, although symptomatic patients had a higher risk of hospitalization [15,16].

Regarding stroke, the absence of differences between symptomatic and asymptomatic patients is consistent with observations from several contemporary registries. Recent studies suggest that symptom status does not substantially modify thromboembolic risk when anticoagulation is appropriately prescribed according to baseline risk (CHA_2_DS_2_-VASc score) [3,17]. This point is particularly relevant, as asymptomatic patients may be undertreated or not anticoagulated because of a perceived lower clinical risk—a tendency also described in EORP-AF and ORBIT-AF [4,16]. Thus, our findings suggest that the more favorable outcomes observed in asymptomatic patients are more likely related to a lower burden of structural and functional heart disease at baseline rather than to the absence of symptoms per se, reinforcing that lack of symptoms should not be interpreted as synonymous with low risk.

Another relevant finding of our study was the lower use of rhythm control strategies in asymptomatic patients, a pattern previously reported in other cohorts [3]. This clinical approach largely reflects current guideline recommendations, which have traditionally prioritized symptom relief as the main indication for rhythm control interventions [6,7]. These recommendations are primarily based on trials that included only symptomatic patients [18,19]. However, studies such as EAST-AFNET 4 have demonstrated that the benefits of early rhythm control are independent of symptom status [5,20]. In the subanalysis by Willems et al., a similar reduction in the composite risk of adverse outcomes was observed in both symptomatic and asymptomatic patients treated with early rhythm control. These findings support the concept that AF represents a progressive disease with prognostic implications beyond symptom burden and suggest that rhythm control strategies should not necessarily be deferred until symptoms develop. However, our study was not designed to establish a causal relationship between earlier rhythm control and improved outcomes in asymptomatic patients. The lower use of rhythm control strategies in this group, together with similar recurrence rates once treatment was implemented, should be regarded as hypothesis-generating rather than definitive evidence supporting earlier intervention; this specific question would require confirmation in a dedicated prospective or randomized study enrolling asymptomatic patients.

Although symptomatic patients received rhythm control strategies, catheter ablation, electrical cardioversion, and antiarrhythmic drugs more frequently than asymptomatic patients, they nonetheless experienced a higher incidence of the composite endpoint. This pattern argues against treatment differences being the primary explanation for the observed outcome disparity, since more intensive rhythm control management would be expected to attenuate, rather than widen, this difference. Treatment strategy was not included as a covariate in the multivariable model, as current guidelines prioritize rhythm control largely on the basis of symptom status, making treatment allocation a plausible mediator rather than an independent confounder on the causal pathway between symptoms and outcomes; adjusting for a mediator could have introduced over-adjustment bias.

Finally, in our cohort, no significant differences in AF recurrence rates were observed between symptomatic and asymptomatic patients after implementation of rhythm control strategies. This finding indicates that symptom status alone does not predict mid-term arrhythmic outcomes and supports the need to consider additional factors when selecting candidates for more aggressive rhythm maintenance therapies. Moreover, given that approximately 40% of AF recurrences after ablation are asymptomatic, these results highlight the importance of considering monitoring strategies beyond symptom-based follow-up in selected patients [21,22].

From a clinical perspective, these findings suggest that asymptomatic atrial fibrillation should not be considered a low-risk condition per se. Although asymptomatic patients exhibited lower cardiovascular mortality, the incidence of major clinical events was not negligible, and outcomes such as stroke, heart failure worsening, and arrhythmia recurrence were comparable to those observed in symptomatic patients. Therefore, management decisions should be guided by overall cardiovascular risk and comorbidity burden rather than symptom status alone. In addition, the lower use of rhythm control strategies in asymptomatic patients highlights a potential gap between evidence and clinical practice, reinforcing the need for individualized treatment strategies that consider disease progression and prognostic implications beyond symptom relief.

## 5. Limitations

Several limitations should be acknowledged. First, this was an observational analysis based on a prospective multicenter registry; therefore, causal relationships between symptom status, therapeutic strategies, and clinical outcomes cannot be established. Treatment decisions—including rhythm control, anticoagulation, and interventional procedures—were left to the discretion of the treating physician, introducing potential selection bias while also reflecting the heterogeneity of contemporary real-world clinical practice. Treatment strategy (rhythm control, ablation, cardioversion, and antiarrhythmic drug use) also differed between groups and was not included as a covariate in the multivariable model, given its plausible role as a mediator rather than a confounder in the relationship between symptom status and outcomes; this approach may nonetheless have limitations inherent to observational data. Second, symptomatic patients exhibited a higher prevalence of cardiovascular comorbidities, including HF, hypertension, cardiomyopathy, and reduced left ventricular ejection fraction. Consequently, the observed differences in cardiovascular outcomes may have been substantially influenced by baseline comorbidity burden rather than symptom status itself. Although multivariable adjustment was performed using clinically relevant covariates, residual confounding cannot be excluded. In addition, propensity score matching, inverse probability weighting, and sensitivity analyses excluding patients with HF or reduced left ventricular ejection fraction were not performed; given the sample size of this subanalysis and the limited number of events for individual secondary endpoints, these approaches could have reduced statistical power and precision. In addition, the proportional hazards assumption for the multivariable Cox model was not formally tested. These analyses represent an important direction for future studies using the REGUEIFA registry with larger sample sizes. Third, AF-related symptoms may not exclusively reflect arrhythmia burden itself, but also concomitant structural heart disease, HF, impaired functional status, or other non-arrhythmic comorbidities. Therefore, differentiating whether symptoms were directly attributable to AF or to associated clinical conditions remains challenging. Fourth, symptom status was assessed according to EHRA classification at predefined study visits, without systematic continuous assessment throughout follow-up. The marked increase in the proportion of asymptomatic patients during follow-up likely reflects the dynamic nature of AF-related symptoms, including symptom improvement after treatment, adaptation to arrhythmia, or changes in rhythm status over time. This dynamic change was not incorporated into the statistical models, which relied on baseline symptom status; a time-dependent Cox model incorporating updated EHRA status throughout follow-up was beyond the scope of the present analysis and represents an important direction for future research using the REGUEIFA registry. Finally, because the REGUEIFA registry included patients managed in cardiology outpatient clinics, these findings may not be directly generalizable to screening-detected, device-detected, or non-cardiology AF populations. In addition, AF recurrence was assessed according to routine clinical practice without systematic continuous rhythm monitoring, potentially underestimating asymptomatic recurrences.

## 6. Conclusions

In this contemporary real-world registry, asymptomatic atrial fibrillation patients exhibited a lower burden of cardiovascular comorbidities and lower cardiovascular mortality than symptomatic patients, whereas rates of stroke, worsening heart failure, major bleeding, and arrhythmia recurrence were similar between groups. These findings suggest that differences in prognosis may be influenced more by baseline cardiovascular risk profile and comorbidity burden than by symptom status alone. Accordingly, asymptomatic AF should not be considered a low-risk condition, and management strategies should be guided by overall cardiovascular risk assessment beyond symptom burden alone. The independent association between symptomatic status and adverse outcomes, although statistically significant, was modest and should be interpreted cautiously in light of the observational design and the potential for residual confounding.

## Figures and Tables

**Table 1:** Baseline characteristics of the study cohort.

Variable	Total (n = 997)	Asymptomatic (n = 336)	Symptomatic (n = 661)	*p* Value
Age (years), mean ± SD	67.61 ± 11.98	67.02 ± 12.62	67.91 ± 11.65	0.478
Female sex, n (%)	321 (32.20)	90 (26.79)	231 (34.95)	0.009
Hypertension, n (%)	622 (62.39)	183 (54.46)	439 (66.41)	<0.001
Diabetes mellitus, n (%)	189 (18.96)	61 (18.15)	128 (19.36)	0.645
Dyslipidemia, n (%)	481 (48.24)	165 (49.11)	316 (47.81)	0.698
Current smoker, n (%)	86 (8.63)	34 (10.12)	52 (7.87)	0.231
Chronic kidney disease, n (%)	64 (6.42)	19 (5.65)	45 (6.81)	0.483
COPD, n (%)	107 (10.73)	32 (9.52)	75 (11.35)	0.379
Sleep apnea, n (%)	50 (5.02)	14 (4.17)	36 (5.45)	0.382
COPD or sleep apnea, n (%)	145 (14.54)	45 (13.39)	100 (15.13)	0.462
Dementia, n (%)	7 (0.70)	3 (0.89)	4 (0.61)	0.694
Anemia, n (%)	40 (4.01)	10 (2.98)	30 (4.54)	0.235
Malignancy, n (%)	83 (8.32)	24 (7.14)	59 (8.93)	0.335
Heart failure, n (%)	148 (14.84)	34 (10.12)	114 (17.25)	0.003
Coronary artery disease, n (%)	115 (11.53)	42 (12.50)	73 (11.04)	0.496
Valvular heart disease, n (%)	114 (11.43)	33 (9.82)	81 (12.25)	0.254
Cardiomyopathy, n (%)	78 (7.82)	15 (4.46)	63 (9.53)	0.005
Any thromboembolic event, n (%)	61 (6.12)	17 (5.06)	44 (6.66)	0.320
Ischemic stroke, n (%)	29 (2.91)	10 (2.98)	19 (2.87)	0.928
Peripheral embolism, n (%)	5 (0.50)	2 (0.60)	3 (0.45)	1.000
Transient ischemic attack, n (%)	18 (1.81)	5 (1.49)	13 (1.97)	0.802
Pulmonary embolism/deep vein thrombosis, n (%)	14 (1.40)	3 (0.89)	11 (1.66)	0.405
Any bleeding event, n (%)	35 (3.51)	11 (3.27)	24 (3.63)	0.772
Alcohol consumption, n (%)	584 (58.58)	196 (58.33)	388 (58.70)	0.912
Obesity, n (%)	408 (40.92)	136 (40.48)	272 (41.15)	0.838
CHA_2_DS_2_-VASc score, mean ± SD	2.36 ± 1.54	2.17 ± 1.57	2.46 ± 1.51	0.003
HAS-BLED score, mean ± SD	0.70 ± 0.78	0.74 ± 0.74	0.69 ± 0.80	0.101
LVEF (%), mean ± SD	55.96 ± 12.75	58.54 ± 11.37	54.65 ± 13.23	<0.001
Normal LVEF (>50%), n (%)	734 (78.76)	267 (85.58)	467 (75.32)	
Mildly reduced LVEF (41–50%), n (%)	89 (9.55)	23 (7.37)	66 (10.65)	
Moderately reduced LVEF (30–40%), n (%)	77 (8.26)	15 (4.81)	62 (10.00)	
Severely reduced LVEF (<30%), n (%)	32 (3.43)	7 (2.24)	25 (4.03)	
Mitral regurgitation, n (%)	104 (10.43)	30 (8.93)	74 (11.20)	0.268
Aortic regurgitation, n (%)	26 (2.61)	6 (1.79)	20 (3.03)	0.246
Mitral stenosis, n (%)	10 (1.00)	2 (0.60)	8 (1.21)	0.509
Aortic stenosis, n (%)	45 (4.51)	11 (3.27)	34 (5.14)	0.179
First AF diagnosis, n (%)	377 (37.81)	149 (44.35)	228 (34.49)	0.002

**Table 2:** EQ-5D quality-of-life questionnaire.

	Total	Asymptomatic	Symptomatic	*p* Value
Mobility problems, n (%)	288 (30.93)	68 (22.08)	220 (35.31)	<0.001
Self-care problems, n (%)	75 (8.06)	21 (6.82)	54 (8.67)	0.329
Problems with usual activities, n (%)	214 (22.99)	46 (14.94)	168 (26.97)	<0.001
Pain/discomfort, n (%)	286 (30.72)	79 (25.65)	207 (33.23)	0.018
Anxiety/depression, n (%)	281 (30.18)	73 (23.70)	208 (33.39)	0.002
Visual Analog Scale score, mean ± SD	68.48 ± 16.25	72.26 ± 13.81	66.61 ± 17.03	<0.001

**Table 3:** Incidence rates of the composite endpoint and individual secondary endpoints at 1 and 2 years. Rates are expressed per 100 person-years.

Endpoint	Person-Years	Events	Incidence Rate	95% CI	Rate Ratio	95% CI	*p* Value
**All-cause mortality**							
Asymptomatic	727.00	18	2.48	1.56–3.93	Ref.		
Symptomatic	1402.15	58	4.14	3.20–5.35	1.67	0.99–2.84	0.055
**Cardiovascular mortality**							
Asymptomatic	727.00	5	0.69	0.29–1.65	Ref.		
Symptomatic	1402.15	30	2.14	1.50–3.06	3.11	1.21–8.02	0.013
**Ischemic stroke**							
Asymptomatic	727.00	3	0.41	0.13–1.28	Ref.		
Symptomatic	1402.15	10	0.71	0.38–1.33	1.73	0.48–6.28	0.400
**Major bleeding**							
Asymptomatic	727.00	20	2.75	1.77–4.26	Ref.		
Symptomatic	1402.15	61	4.35	3.38–5.59	1.58	0.95–2.62	0.073
**Worsening heart failure**							
Asymptomatic	727.00	44	6.05	4.50–8.13	Ref.		
Symptomatic	1402.15	115	8.20	6.83–9.85	1.36	0.96–1.92	0.085
**Composite endpoint**							
Asymptomatic	727.00	61	8.39	6.53–10.78	Ref.		
Symptomatic	1402.15	170	12.12	10.43–14.09	1.45	1.08–1.94	0.013

**Table 4:** Multivariable Cox regression analysis for the composite endpoint of cardiovascular death, worsening heart failure, stroke, and major bleeding.

Variable	Hazard Ratio	95% CI	*p* Value
Symptomatic vs. asymptomatic AF	1.40	1.02–1.91	0.036
Female vs. male sex	0.82	0.60–1.11	0.191
Cardiomyopathy	0.69	0.43–1.09	0.115
Hypertension	0.71	0.49–1.02	0.065
CHA_2_DS_2_-VASc score	1.58	1.41–1.76	<0.001
LVEF ≤ 40%	1.98	1.39–2.83	<0.001

## Data Availability

The data that support the findings of this study are available from the corresponding author upon reasonable request and with permission of the REGUEIFA registry steering committee.
